# Pediatric asthma and autism—genomic perspectives

**DOI:** 10.1186/s40169-015-0078-x

**Published:** 2015-12-14

**Authors:** Sunghee Oh, Hong Ji, Drew Barzman, Ping-I Lin, John Hutton

**Affiliations:** Department of Computer Science and Statistics, Jeju National University, Jeju Ciy, Jeju Do South Korea; Division of Asthma Research, Department of Pediatrics, Cincinnati Children’s Hospital Medical Center, Cincinnati, OH 45229 USA; Division of Psychiatry, Department of Pediatrics, Cincinnati Children’s Hospital Medical Center, Cincinnati, OH 45229 USA; Division of Biostatistics and Epidemiology, Department of Pediatrics, Cincinnati Children’s Hospital Medical Center, Cincinnati, OH USA; Division of Biomedical Informatics, Department of Pediatrics, Cincinnati Children’s Hospital Medical Center, Cincinnati, OH 45229 USA

**Keywords:** Genomic variation, Transcription, Pathobiology, Pediatric disorders, Autism, Asthma

## Abstract

High-throughput technologies, ranging from microarrays to NexGen sequencing of RNA and genomic DNA, have opened new avenues for exploration of the pathobiology of human disease. Comparisons of the architecture of the genome, identification of mutated or modified sequences, and pre-and post- transcriptional regulation of gene expression as disease specific biomarkers are revolutionizing our understanding of the causes of disease and are guiding the development of new therapies. There is enormous heterogeneity in types of genomic variation that occur in human disease. Some are inherited, while others are the result of new somatic or germline mutations or errors in chromosomal replication. In this review, we provide examples of changes that occur in the human genome in two of the most common chronic pediatric disorders, autism and asthma. The incidence and economic burden of both of these disorders are increasing worldwide. Genomic variations have the potential to serve as biomarkers for personalization of therapy and prediction of outcomes.

## Introduction

Both autism spectrum disorder (ASD) and asthma are among the most common pediatric chronic diseases worldwide and pose an enormous economic burden on health care delivery systems [1, 2]. There has been recent progress in identifying underlying genomic changes in these disorders, which will be reviewed here. Unveiling changes in the genome that underlie disease identify new biomarkers that have the potential to predict outcomes of treatment and to identify targets for development of new therapies.

## Review

### Copy number variation

Copy number variants (CNVs) are structural alterations of DNA that increase or decrease the number of copies of one or more sections in a strand of DNA within a chromosome [3]. CNVs have been associated with certain diseases, for example autism spectrum disorder (ASD) and schizophrenia. Family and twin studies have strongly suggested that certain genetic variants can substantially influence the risk of ASD and *de novo* mutations in the form of CNVs contribute to the risk of autism [4, 5]. Recent deep sequencing studies have generated robust evidence for the link between recurrent rare variants and ASD [6–8]. Vaishnavi et al. found that 11 % of ASD-related copy number variants (CNVs) contained microRNAs (miRNAs) [9]. These CNV-miRNAs formed a regulatory loop with transcription factors and their downstream target genes, and annotation of these target genes indicated their functional involvement in neurodevelopment and synapse [10]. miRNA studies provide mechanistic insights into genetic regulations that may modulate the risk of ASD. These findings exemplify how functional genomic approaches may facilitate the efforts to decipher genetic mechanisms underlying clinical features of ASD and other heritable disorders. However, susceptibility genes for ASD remain elusive because of difficulty in replicating linkage or association findings [11].

The presence of CNV in childhood asthma has only been recently discovered. Rogers et al. showed that CNV in candidate asthma genes was prevalent in 383 asthmatic trios participating in the Childhood asthma management program (CAMP) [12]. However, the vast majority of identified CNVs were of rare frequency (<5 %) and were not statistically associated with asthma. 2 CNVs near NOS1 and SERPINA3 were modestly association with asthma and they were unlikely to explain the previously identified associations between SNP and asthma.

### Gene fusion

While gene fusions are well known as a major factor in both initiation and progression of cancers [13], they are rare in both diseases in our study. Copy number variants are known to occur in both ASD and asthma. CNVs can facilitate combining parts of two genes, resulting in a fusion transcript. Holt et al. [14] identified fusion-gene generating CNVs in probands with ASD, however there was no difference in overall frequency of fusion transcripts between patients and normal controls. Ceroni et al. [15] identified a child with both ASD and asthma, who inherited a maternal deletion that resulted in a BST1-CD38 fusion transcript. Whether the fusion transcript causes changes in function that underlies ASD and/or asthma in the child has not yet been studied.

### Non-coding RNAs

Non-coding RNAs, both microRNAs (miRNA) and long noncoding RNAs (lncRNA), play a key role in functional genomics—stability and maintenance of gene expression. For example, they both play a major role in regulating chronic inflammatory diseases such as asthma [16]. Geaghan and Cairns have reviewed evidence that numerous microRNAs (miRNA) play a role in ASD and other psychiatric disorders, characterized by dysregulation of target transcripts [17]. Abnormal large noncoding RNAs significantly contribute to the pathology of autistic brain [18, 19].

### Mutations in mitochondrial genome

Mitochondrial dysfunctions occur in several human inherited disorders [20]. Mitochondrial dysfunction is hypothesized to play a role in asthma. Flaquer et al. compared mitochondrial SNPs in 372 asthmatic children and 395 health controls [21]. Different variants were found in asthmatic boys and girls. For boys significant differences were found in the CYB gene; for girls in the NADH-dehydrogenase subunits. Post-mortem brain studies have suggested an elevated prevalence of mitochondrial dysfunction in ASD [22].

### Epigenetic modification of the genome

Expression of genes can be turned on or off or modified by factors other than an individual’s DNA sequence. Although all organs of the body contain the same DNA, the pattern of expression of genes differs from organ to organ because of epigenetic factors [23]. The most common modification that influences expression of genes is DNA methylation. Methylation of DNA involves the conversion of cytosine to methylcytosine at a site in DNA. The cytosine nucleotide to be methylated is located next to a guanine nucleotide, i.e. in a CpG dinucleotide, although recent research has found methylated cytosine in other sequence contexts, such as CpA [24]. Genes with highly methylated promoters typically are not well expressed. Changes from normal patterns of DNA methylation of specific genes can cause alterations in gene expression that are associated with disease. Epigenome-wide association studies (EWAS) hold promise for the detection of new regulatory mechanisms that may be susceptible to modification by environmental and lifestyle factors affecting disease [25]. Because of the rapid advances in sequencing technology, large numbers of methylated CpG sites can be identified across the entire genome. Because DNA methylation is tissue-specific, having a mixed cell population makes it hard to link the observed DNA methylation patterns to the disease rather than to changes in cell populations. Investigators must prospectively identify the sources of cells and define appropriate profiling methods.

The role of DNA methylation in pediatric diseases has recently been established. Most of the data are derived from studies of genes the investigators selected because they were thought to be involved in causation of the disease, the candidate gene approach. With the emergence of high-throughput technologies, ranging from microarrays to next-gen sequencing, genome-wide scanning to search for disease-related DNA methylation markers is now possible. The interplay among DNA methylation sites, genetic variation, protein binding sites, and gene expression is a very active field of investigation.

Emergent evidence has shown that epigenetic modifications of DNA, rather than single-locus variation, may account for the heritability of ASD [26–28]. Compared to other psychiatric disorders with variable onsets across the lifespan, ASD, where the onset occurs by the age of two, represents one of the few disorders in which time-dependent penetrance is not a primary concern. The high heritability of ASD may also lead to a higher chance of success in mapping the risk genes for this disorder. Prevalence of ASD has a male excess of 4:1 and dysregulation of methylation in brain-expressed genes on the X-chromosome has been speculated to contribute to the development of ASD [29]. Nararajan and colleagues reported that a significant reduction in the expression of the *MeCP2* gene, which encodes methyl CpG binding protein 2, was found in 79 % of frontal cortex samples of patients with ASD, and increased *MeCP2* promotor methylation was particularly prominent in males with ASD compared to controls [30]. A recent study by Ladd-Acosta et al. [31] investigated over 485,000 CpG loci across functionally relevant genomic regions using the Infinium Human Methylation 450 Bead Chip and identified 4 differentially methylated regions located within *PRRT1*, *TSPAN32*, *C11orf21*, *ZFP57* and *SDHAP3* from different parts of the brain, which offers novel candidate genes for ASD. Genomic imprinting, which arises from parent-specific methylation patterns, has been thought to play a role in ASD because of findings of parent-of-origin effect on some chromosomal regions, such as 7q. It has been proposed that ASD is caused by an overbalance of paternally expressed genes. Schneider and colleagues analyzed the methylation and expression patterns of the *MEST*, *COPG2*, and *TSGA14* on 7q in the brain cortex of humans and a variety of primates [32]. Compared to other primates, expression of the *COPG2* gene was down regulated in human cortex because of methylation. The authors suggest that down regulation of *COPG2* in humans may partially account for the emergence of the more advanced “social brain”. ASD is considered a disorder of the “social brain”, therefore genetic and epigenetic variants in genes such as *COPG2* may play a role in susceptibility to autism and other pediatric mental disorders [33].

Stefanowicz et al. studied DNA methylation in airway epithelial cells (AECs) and peripheral blood mononuclear cells (PBMCs) from atopic, atopic asthmatic, non-atopic asthmatic children and healthy controls [34]. To measure genomic methylation they used Illumina Golden Gate Methylation Cancer Panel 1, which includes 1505 CpG loci across 807 genes. Gene expression was performed using RT-PCR. Of the differentially methylated CpG sites in airway epithelial cells, 13 were specific to healthy controls, 8 were found only in atopics, and 6 were unique to asthmatics. Genomes from asthmatics differed from atopics at 8 sites that included CpG sites in genes that encode transcription factor STAT5A and zinc transport protein CRIP1. The authors found that *STAT5A* gene expression is decreased and *CRIP1* expression is elevated in airway epithelial cells from asthmatics compared to healthy and atopic subjects. In PBMCs no differences in the methylation status of these genes were found, so that PBMCs did not serve as alternatives to studies of airway epithelial cells. This study highlights the importance of studying the right tissue, when identifying epigenomic changes in disease. A recent study by Yang et al. [35] compared the blood DNA methylation levels at ~ 480,000 CpG sites between 97 controls and 97 asthmatic patients and identified 81 differentially methylated CpG sites. Validated CpG sites are located at *RUNX3*, *IL4*, and *catalase*. CpG sites associated with serum IgE among asthmatics were also discovered. Studies of DNA methylation are often coupled with gene expression studies and genetic variation studies, as DNA methylation can regulates gene expression [36] and SNPs also modifies DNA methylation [37, 38]. Acevedo and colleagues studied the association of childhood asthma with CpG sites polymorphisms, regional DNA methylation and gene expression at the *GSDMB/ORMDL3* locus, which is located at 17q21, a novel asthma-susceptibility locus found in ethically diverse populations [39]. As expected, the CpG sites polymorphisms that either create or remove CpG sites alters DNA methylation and are associated with asthma. They are also associated with mRNA expression changes in *ORMDL3*. In addition, the methylation levels at *ORMDL3* promoter in asthmatic children is also significantly higher compared to controls, and is correlated with *ORMDL3* expression in blood leukocytes. Interestingly, SNPs and CpG methylation are independently associated with *ORMDL3* expression, suggesting two independent mechanisms regulating gene expression.

### Gene expression profiles

Exploring gene expression profiles of normal and diseased tissues has long played a major role in understanding the pathophysiology of disease. Originally such studies were primarily conducted using expression microarrays. Use of microarrays is now typically coupled with studies using high throughput technologies such as RNA-seq [40–47]. These techniques have enabled the generation of lists of top putative candidates of differentially expressed genes between different groups in diseases of interest. Studies of gene expression generate information about alternative splicing of RNA transcripts, the role of non-coding genomic elements (ncRNAs, ncDNAs, and microRNAs), and epigenetic changes in the genome [48–51].

Hundreds of susceptibility loci and candidate genes for ASD have been identified [52, 53]. Despite these findings, only a handful of genetic variants have been consistently found to be associated with the risk of ASD across different population. Whole-exome sequencing has identified biallelic mutations in several genes previously associated with ASD (*AMT, PEX7, SYNE1, APS13B, PAH*, and *POMGNT1*) and demonstrated the importance of partial loss of gene function in this disorder [54]. Splicing mutations may also play a role in susceptibility to ASD [55–57]. Yan and colleagues [58] found that neurexin 1alpha structural variants, including a splicing mutation, could distinguish patients with ASD from healthy controls. Genetic variation in ion channel genes, such as *CACNA1C* and *CADPS2*, may modulate the risk of ASD [59–61]. A genome-wide scan of micro RNAs (miRNAs) in cell lines established from patients with ASD compared to normal controls suggested that miRNAs dysregulated several coding and non-coding genes, including *HEY1, SOX9, miR*-*486* and *miR*-*181b*, and increased the risk of ASD [62]. All of these genes are involved in nervous system development and function. One recent study of postmortem human brains reported that noncoding antisense RNA transcripts are generated at approximately 40 % of loci previously implicated in ASD. The antisense RNA corresponding to synaptic ras GTPase-activating protein 1 (SYNGAP1) was differentially expressed in brain regions from patients with ASD compared to normal controls [63].

Microarray and RNA-sequencing experiments have been performed on nasal epithelial cells, bronchial epithelial cells, small airways, and blood cells from asthmatics and controls [64–68]. Significant differential expression in 70 genes in nasal cells, including *IL13, IL5*, periostin (*POSTN*), calcium-activated chloride channel regulator 1 (*CLCA1*), and serpin peptidase inhibitor, clade B (*SERPINB2*), was found to be linked to airway remodeling, production of mucus, and shifting of the immune response toward the Th2 phenotype thus enhancing asthma exacerbation [67, 68]. Genome-wide association studies have identified *CDHR3* (cadherin-related family member 3) gene as a novel susceptibility locus for early life childhood asthma with severe exacerbations [69]. This gene is highly expressed in airway epithelium and encodes a calcium-dependent cell adhesion protein. Custom microarrays designed to measure expression of genes thought to play a role in the pathogenesis of asthma have proved useful in establishing the biological relevance of these genes [70]. *KLF3A* was identified as a susceptibility gene for childhood asthma by gene expression arrays in nasal cells and further studies found that genetic variation in *KLF3A* is associated with asthma.

### Genetic regulatory networks

Information on co-regulators and co-expression of genes in normal and disease states has increased interest in genetic regulatory networks. The generation of genetic variation/gene expression profiles by either array or sequencing platforms affords the opportunity to define connectivity maps. This permits assignment of genetic variants associated with pediatric disorders to specific biochemical pathways and regulatory networks [71–73]. These technologies have been applied to ASD, cancer and other diseases and have uncovered mechanisms of action of disease specific target genes. Knowledge of genomic changes can be used to identify the biochemical pathways and regulatory networks that are affected and identify potential targets for drug therapy. Molecular classification of a patient’s disease should improve choices of therapy at diagnosis, given a particular molecular subtype, and lead to development of new therapies for subtypes with a poor prognosis.

## Conclusions

Besides identifying disease specific biomarkers, one future direction is to integrate the studies in genome architecture, epigenomics, and gene expression to understand the underlining mechanisms of pediatric diseases. Cutting-edge genome/epigenome-editing tools have the potential to correct disease-related variations and to develop novel therapies. We have discussed genomic mutations and epigenetic modifications in pediatric asthma and ASD. Many of these advances represent progress beyond the era of functional genomics, which focused on measurements of gene expression. The products of post-transcriptional and genomic variations, including non-coding genetic elements and copy number variations are drivers of disease initiation and progression. Unveiling changes in the genome that underlie disease identify new biomarkers that should prove to be associated with different outcomes of treatment and provide targets for development of new therapies. The continuing development of robust methods for NGS (next generation sequencing) and other technologies will continue to improve our ability to identify causes of disease in an individual patient and to use this information to personalize therapy.
